# Evaluation of Antioxidant Potential of *Kaempferia rotunda* Linn

**DOI:** 10.4103/0250-474X.43002

**Published:** 2008

**Authors:** J. Priya Mohanty, L. K. Nath, Nihar Bhuyan, G. Mariappan

**Affiliations:** Himalayan Pharmacy Institute, Majhitar, Rangpo, East Sikkim-737 132, India

**Keywords:** Lipid peroxidation, antioxidants, malonaldehyde, 4-hydroxyl-2-nonenal, *Kaempferia rotunda* Linn

## Abstract

The plant *Kaempferia rotunda* Linn. has been explored for its anti oxidant potential in the present study. The antioxidant property was assessed by lipid peroxidation markers such as malonaldehyde (MDA) and 4-hydroxyl-2-nonenal (4-HNE). The lipid peroxidation byproducts are highly toxic and responsible for various diseases like myocardial infarction, diabetes mellitus, hepatic injury, atherosclerosis, rheumatoid arthritis and cancer. The chemical constituents of the plant were critically and qualitatively analyzed to confirm the presence of flavonoids and phenolic derivatives. Hence our objective has been designed to evaluate the antioxidant effect of *Kaempferia rotunda* linn. and its contribution to control the lipid peroxidation.

The free radicals such as hydroxyl radicals (OH^-^), superoxide radicals (O_2_^-^), singlet oxygen (O^-^) and hydrogen peroxide radicals (H_2_O_2_) play a significant role in age dependent diseases such as atherosclerosis, myocardial infarction, diabetes mellitus, rheumatoid arthritis and cancer^1^,^2^. The free radicals deliberately activate the lipid peroxidation^3^. Hence various toxic metabolites are generated i.e. MDA and 4-HNE. The malonaldehyde can attack NH_2_group of protein molecules to form both intramolecular and intermolecular cross links between different proteins causing severe damage to membrane proteins. These metabolites have been associated with damaging effects of oxidative stress, oxygen toxicity and liver injury^4^.

The plant *Kaempferia rotunda* linn. belongs to the family Zingiberaceae also named *bhuichampaka* (Sanskrit), *bhuchampa* (Hindi) and blackhorm (English). It is a fragrant aromatic herb with a tuberous rhizome distributed throughout India^5^. In some districts of Maharashtra the powder root is popular in mumps and also said to be used in the form of poultice, promotes suppuration. The main constitutent crotepoxide is useful for the inhibition of tumors^6^.

Phytochemically the plant has been attributed to contain flavonoids, crotepoxide, chalcones, quercetin, flavonols, β-sitosterol, stigmosterol, syringic acid, protocatechuic acid and some hydrocarbons have been previously reported^7^. The recent literature review revealed that abundant presence of flavonoids in the plant of interest plays prime role in antioxidant mechanisms^8^–^10^.

The tuberous rhizomes of *Kaempferia rotunda* Linn were collected during July-August from the various areas of Sikkim Himalayan region and authenticated by Botanical Survey of India, Gangtok, Sikkim. The dried, powdered rhizomes were subjected to Soxhlet extraction successively using methanol. The extract was filtered, concentrated in vacuum under reduced pressure. The yield value was found to be 8.5%. The extract was subjected to qualitative chemical investigation for phytochemical constituents like flavonoids, steroids, triterpenoids and crotepoxide.

The liver was chosen to estimate the markers of lipid peroxidation because the metabolism of toxic metabolites and free radicals occur mainly in liver. The metabolites from liver may diffuse into various extra hepatic tissues causing lipid peroxidation and cellular damage^11^.

The fresh goat liver was obtained from local market, stored in phosphate buffer, homogenized (1g/ml) and filtered to get clear homogenate. The lipid peroxidation indicator i.e. MDA was estimated using thiobarbituric acid reacting substances (TBARS) by the method of Ohkawa *et al*^12^. The lipid peroxidation was induced in goat liver homogenate by ferrous sulphate. The generated MDA reacts with thiobarbituric acid at pH 3.5, produces a pink coloured complex, which has λmax at 530 nm. The concentration of MDA was calculated by calibration of standard graph through regression method. The significance of the results was analyzed by statistical method.

The liver supernatant was mixed with 2,4-dinitrophenylhydrazine (DNPH) and incubated for 1 h at room temp. The formed adduct of 4-HNE and DNPH was measured at 350 nm in UV/Vis spectrophotometer^13^. The quantity of 4-HNE was calculated by linear regression analysis. The data were expressed as mean±SE differences between groups were analyzed using one way analysis of variance (ANOVA) followed by Bonferroni multiple range test. The results are statically significant at p <0.05 levels.

The experimental study was based on the estimation of MDA and 4-HNE and their suppression by *Kaempferia rotunda* Linn. and presented in Table 1. From the experimental results, it proved that the methanol extract of the plant has significant antioxidant property. The quantification of MDA and 4-HNE can be directly correlated with the lipid peroxidation inhibition capacity of the extract. The antioxidant property was studied for dose dependency. From the fig. 1 it was concluded that the antioxidant property has inverse relationship with dose i.e. high at low dose and vice versa. The extract 100 μg/ml and 200 μg/ml have significant and moderate antioxidant property; respectively but 500 and 1000 μg/ml has insignificant property. The antioxidant property has inverse relationship; it may be due to the presence of crotepoxide, which increases the peroxidation.

**TABLE 1:** *IN VITRO* EFFECT OF *KAEMPFERIA ROTUNDA* ON LIPID PEROXIDATION

Concentration of extract (μg/ml)	MDA (μM)	4-HNE (nM)
Control	18.87±0.15	97.59±0.18
Ascorbic acid	13.57±0.22	75.82±0.16
100	14.44±0.21	82.90±0.23
200	16.86±0.15	88.98±0.14
500	17.29±0.10	93.58±0.28
1000	17.95±0.08	95.09±0.13

Each value is the mean concentration ± standard error of the mean. All values are significant at P<0.05

**Fig. 1:**
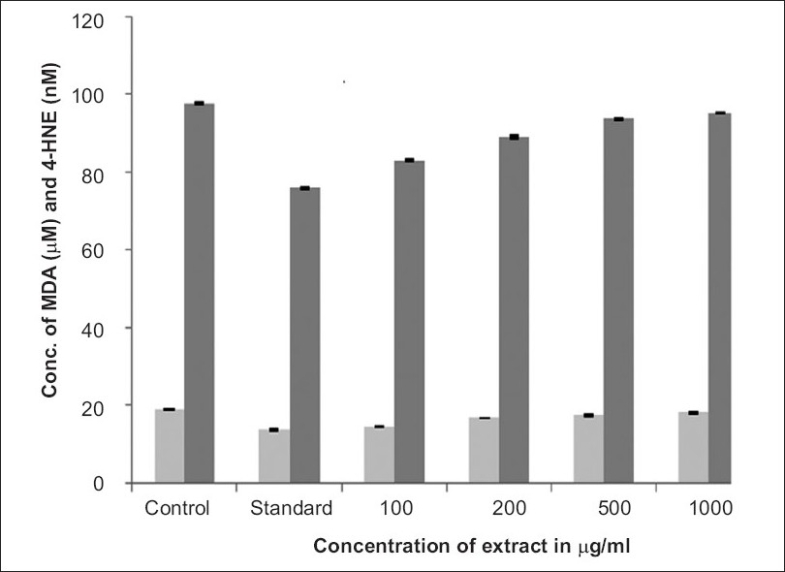
MDA and 4-HNE scavenging activities of *Kaempferia rotunda* Each bar represents the average of three determinations with standard error. The probability levels of changes are significant at P<0.05. Concentration of MDA in μM (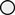) and concentration of 4-HNE in nM (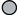)

At low dose the level of crotepoxide is low hence peroxidation is less and the flavanoids can scavange all the radicals. As a result the antioxidant property is more but in higher dose the total phenomena is reversed. The antioxidant property of extract was compared with standard antioxidant (ascorbic acid). The scavenging of the radicals by the methanol extract of *Kaempferia rotunda* in the above study has thus been correlated with the antioxidant potential of the plant and this information can be used to control the age dependent diseases mentioned above.

## References

[1] Visioli F, Borsani L, Galli C (2000). Diet and prevention of coronary heart disease: The potential role of Phytochemicals. Cardiovasc Res.

[2] Breimer LH (1990). Molecular mechanism of oxygen radical carcinogenesis and mutagenesis: The role of DNA base damage. Mol Carcinog.

[3] Halliwell B, Gutteridge JM (1986). Iron and free radical reactions: Two aspects of antioxidant protection. Trends Biochem Sci.

[4] Adkinson D, Hollwarth ME, Benoit JN, Parks DA, Granger DN (1986). Role of free radicals in ischemia-reperfusion injury to the liver. Acta Physiol.

[5] Gurung B (2002). The Medicinal plants of Sikkim Himalaya.

[6] Kupchan SM, Hemingway RJ, Smith RM (1969). Crotepoxide, a novel cyclohexane diepoxide tumor inhibitor from Croton macrostachys. J Org Chem.

[7] Pai BR, Rao NN, Wariyar NS (1970). Occurrence of crotepoxide in *Kaempferia rotunda* linn. Indian J Chem.

[8] Sirat HN, Jamil S, Siew LW (2001). Constituents of *Kaempferia rotunda* linn. Chem Res Commun.

[9] Pietta PG (2000). Flavonoids as antioxidants. J Nat Prod.

[10] Middleton E (1984). The flavonoids. Trends Pharmacol Sci.

[11] Ames BN (1989). Endogeneous oxidative DNA damage, aging and cancer. Free Radic Res Commun.

[12] Okhawa H, Ohishi N, Yagi K (1979). Assay of lipid peroxide in animal tissue by thiobarbituric acid reaction. Anal Biochem.

[13] Kinter M, Punchard NA, Kelly GJ (1996). Free radicals: A practical approach.

